# “Co-production Compass” (COCO): An Analytical Framework for Monitoring Patient Preferences in Co-production of Healthcare Services in Mental Health Settings

**DOI:** 10.3389/fmed.2020.00279

**Published:** 2020-07-03

**Authors:** Guendalina Graffigna, Serena Barello, Lorenzo Palamenghi, Fabio Lucchi

**Affiliations:** ^1^Faculty of Agriculture, Food and Environmental Sciences, Università Cattolica del Sacro Cuore, Piacenza, Italy; ^2^EngageMinds HUB-Consumer, Food & Health Engagement Research Center, Milan, Italy; ^3^Department of Psychology, UniversitÃ Cattolica del Sacro Cuore, Milan, Italy; ^4^Spetali Civili, Brescia, Italy

**Keywords:** co-production, patient input, patient engagement, recovery, mental health, evaluation framework

## Abstract

**Background:** Engaging patients in raising their voices to advocate for their priorities being taken into account is today acknowledged as essential to improve research and decision-making in healthcare. However, literature is scarce regarding an evaluation framework to monitor the extent to which this approach is successful, in particular in mental health, where the application of patient-reported outcome measures (PROMs) is particularly difficult. In this study, we describe the process of development and first implementation of a new assessment framework—“Co-production Compass” (COCO) framework—for monitoring patient preference collection in co-production of healthcare services within the scope of a national-based project (namely, Recovery.Net) in the mental health field.

**Method:** We conducted (1) a narrative scan of relevant scientific literature on patient engagement in service co-production and (2) qualitative analysis of five subsequent workshops involving—in total−144 expert stakeholders (i.e., expert patients, doctors, nurses, psychologists, healthcare managers…). Data analysis involved three phases: identifying the themes, developing a framework, and confirming the framework. We coded and organized the data and abstracted, illustrated, described, and explored the emergent themes using thematic analysis. At the same time, content analysis was conducted to retrieve concepts and insights from relevant literature about health services co-production to integrate and extend the emergent conceptual framework. The framework was finally reviewed by the research partners belonging to the study project and preliminarily implemented.

**Results:** According to the results of both the literature scan and the participatory workshops, the COCO evaluation framework for monitoring patient preference collection when coproducing medical pathways was drafted. The framework comprised of three organizing themes, corresponding to the three code clusters, which emerged from both the stakeholders' workshop data and relevant scientific literature: “*the need for shared and practice-oriented evaluation standards*”; “*the quest for a multi-dominion approach*”; “*the need for a multi-stakeholder evaluation*”. These themes were interconnected and formed a conceptual framework to measure the phenomenon of meaningful patient involvement in healthcare co-production. This framework was endorsed by the research partners of the project and preliminarily applied in a mental health setting.

**Conclusion:** The COCO framework provides guidance on aspects of co-production in healthcare to address for meaningful patient involvement in giving their inputs for more effective service and drug development processes. It could be particularly useful when monitoring patient–researcher partnership initiatives.

## Introduction

Engaging patients in raising their voices to advocate for their priorities being taken into account is acknowledged as essential to improve research and decision-making in healthcare (1–4). Patient preference elicitation and collection, indeed, are becoming a recurrent practice aimed not only at the definition of individual therapeutic plans (5) but also for the development of new drugs and devices (6, 7) and for the co-production of healthcare services (8, 9) in many clinical fields, among which mental health is an interesting observatory for specificities. Traditionally, mental health outcomes have tended to be not only symptom-based but also reflecting the process of service users' recovery in their quality of life. That is why, currently, there is a large and growing impetus among patients' representatives, policy makers, clinicians, and researchers toward the engagement of patients/users of mental health services (and other layperson/non-professional service users in health- and healthcare-related research as well) since it has the potential to improve the feasibility and applicability of routine clinical practices and to increase the effectiveness of drug development processes. For instance, the European Medicines Agency (EMA) has long recognized the importance of incorporating the patients' perspectives in the course of the drug life cycle (10) and within regulatory processes related to decision-making about new drug introduction. Also, there is an overarching ethical imperative for patient engagement in research as a manifestation of the “democratization” of the research process. Authors agree that collecting patients' preferences regarding their healthcare is an important contribution to the formulation of guidelines and care services in mental health. Such guidelines must be flexible to take into consideration the differences between patients regarding their values, choices, and expectations for clinical interventions and outcomes.

This issue has led to a huge amount of research devoted to the development and implementation of rigorous procedures to collect patients' input aimed at improving their care pathways and treatments. In this regard, more and more health organizations have adopted—in their routine practice—systems of continuous monitoring of patient experiences through the collection of patient-reported outcome measures (PROMs) and patient-reported experience measures (PREMs) (11–13), even though full implementation in clinical practice is still far from being achieved (14). These measures are increasingly recognized as a way to systematically collect information about the impact of care interventions on the patients' quality of life and illness management and to collect their preferences about how to more effectively shape the care provision. Not aligning care provision and clinical pathways with patients' perspectives can contribute to patients' non-adherence. Similarly, health interventions that have been developed without taking into account patients' needs and expectations may not be adopted or effectively implemented by patients. Thus, guidelines must not only use the best available research evidence and clinical expertise but should also recognize the added value of service users' involvement (patients, carers, consumers) in scientific research activities such as scientific advisory meetings or collaborative initiatives to address specific matters related to service and drug development, regulatory marketing authorization, and reimbursement decisions [namely, “patient participation in research” (15–17)]. Historically, the role of patients in research ranges from passive (patient as a “data provider”) to active (patient as a “co-researcher”). The recent tradition of democratizing the medical research approach, by involving patients in co-productive processes may be seen as the setting in which patients' input elicitation is maximized and patients assume the most proactive role in research (18). Scientific literature, moreover, is consistent in claiming that engaging patients in sharing their perspective in the co-production of their care paves the way for increased health outcomes, enhanced patient satisfaction, better service innovation, and cost savings (19). Often, the patient perspective is gathered utilizing PROMs. PROMs have the potential to capture outcomes such as sustained symptom reduction, return to functioning, and optimization of patient mental health and recovery.

However, although there is growing interest about the importance of engaging patients in the crucial turning points of the medicine life cycle, as well as in the planning of clinical pathways, there is a lack of evidence and shared methodological frameworks to monitor how and how much patients' preferences are effectively collected along these processes. The systematic elicitation of patients' preferences, indeed, risks to be perceived as an additional burden on the patients' shoulders: this issue is particularly relevant in the mental health setting, where patients may be particularly frail and not equally able to be engaged in giving their preferences, or not in the same entity along all the healthcare or research process. Yet, not every patient wants to participate in healthcare co-production to the same degree (20). Some may wish to be active in providing their inputs but may ultimately want to rely entirely on their psychiatrist to make decisions on their behalf. With the only exception of the (undoubtable) importance of eliciting patient values and inputs for shaping drug development and care pathways, some questions still remain unanswered. How much do people embrace a collaborative model in healthcare? How to sustain patients' willingness to share their preferences along the medicine life cycle? How to support the healthcare system to integrate them in routine organizational practices without burdening patients? This is an undoubtable challenge for the effective implementation of patients' preference collection routines and needs careful consideration and monitoring in order to implement corrective actions and dedicated support. This in particular in the case of mental health patients who may feel vulnerable and may not have the psychological confidence to provide their input. This also implies that patients, to be effectively engaged in such activities, require specific skills, competences, and motivation that enable them to actually participate in such collaborative processes. In the case of cognitive impairment or mental health disease, this becomes even more challenging and needs to be carefully taken into consideration in order to ensure an equal and adequate participation of the patients (21, 22).

However, although general protocols and tools exist to sustain researchers and clinicians in applying a co-productive approach in patients' preference elicitation and collection, to the best of our knowledge, literature is very scarce regarding a framework to monitor the extent to which those approaches were successful in reaching this goal and able to capture a multi-actor perspective on such co-productive practices in mental health.

Furthermore, no clear evidences are currently being provided regarding the extent to which the patients' willingness and psychological readiness to get engaged in the planning of their therapeutic path is taken into account, measured, and respected along the whole process; as mentioned above, due to the challenging time and resource-consuming features of patients' preference communication and collection, the patients' availability and readiness to get engaged in such process should not be taken for granted but should always be challenged and discussed in order to ensure an ethical, equal, and sound approach to this issue. In mental health, patients' input and preferences should be continuously taken into account in order not only to more effectively personalize the therapeutic/pharmacological approach but also to enhance its effectiveness in terms of the whole service care delivery. Indeed, in mental health, the involvement of patients is particularly relevant not just for drug development but also for the whole medical and service development process. Research in mental health settings highlights the importance of service users' involvement in the development and review of their care plans as a key asset to facilitate their recovery (23).

Thus, the development of sound and rigorous approaches devoted to the monitoring of the mental health patients' experience and of their engagement in giving their preferences and priorities is a key aspect to be considered for a highly effective implementation of such procedures. Moreover, the absence of a standardized and rigorous way to evaluate co-production processes in all their complexity and multidimensionality risks to lose intelligence on the intervenient variables that contribute to either the success or the failure of these initiatives, this in particular in sensible settings as mental health.

According to these premises, in this study, we describe the process of development and first implementation of a new assessment framework [“Co-production Compass” (COCO)] aimed at:

- monitoring the extent to which mental health patients feel ready to be engaged in a process of preference elicitation and provision and to which they feel their priorities are successfully being taken into account in the concrete planning of their individual therapeutic pathway;
- assessing the “alignment” and “misalignment” among key stakeholders' perspectives (i.e., the patients, their informal caregivers, the healthcare professionals involved in their care, the healthcare service team) in their evaluation of how much mental health patients' preferences are included in the care planning;
- connecting data about mental health patients' preference monitoring with clinical outcomes already monitored in the standard care process.

In this paper, we describe the development of such evaluation framework. This framework was designed as part of a project named “Recovery.Net” that was generally aimed to codesign service for patients with mental health disorders by engaging them in collaborative labs with their mental health providers and caregivers. The evaluation framework, which is the object of this paper, has been developed for, and adopted by, Recovery.Net to monitor the effectiveness of the services developed. Transferability of this framework to other clinical settings will be also discussed.

## Materials and Methods

### The Research Setting: The Recovery.Net Project

The evaluation framework presented in this work was developed within the scope of a national-based project (namely, Recovery.Net) funded by Fondazione Cariplo and aimed to sustain an organizational change related to mental health services in Italy. In particular, the project is devoted to the acquisition of participation and co-productive skills by patients that will be used along their personal journey to enhance their own recovery. In order to achieve this goal, the Recovery.Net project is specifically designed to embrace methodological protocols and co-production tools (such as collaborative workshop, co-created patient profiles, the recovery journey map alternating the patient and professionals' views on the service provision, the value cards providing hints for the rethinking of services, or the inspiring case cards providing tangible examples (24–26) in order to guarantee the effective and equal elicitation of patient preferences along the whole spectrum of their therapeutic pathway.

The project called for an evaluation framework capable of providing systematic evidences of the effective participation and co-productive process between mental health patients, clinicians, and the other key stakeholders in the definition of the individual medical plan. Hence, efforts have been made to develop a tailored evaluation framework able to monitor the extent to which patients are able to effectively express their preferences and participate to the co-production process and to implement it inside the other project activities.

Informed by scientific evidence and perspectives of patients, caregivers, and health professionals, an evaluation framework for monitoring co-production practices in mental health services was developed. In particular, we conducted a scan of the literature on co-production in mental health in parallel with focus groups with key stakeholders (patients, families, and health professionals) in order to get insights about the key issues related to effective service co-production.

In this paper, we shall describe the framework developed and adopted in the Recovery.Net project.

### Procedures

**Figure 1:**
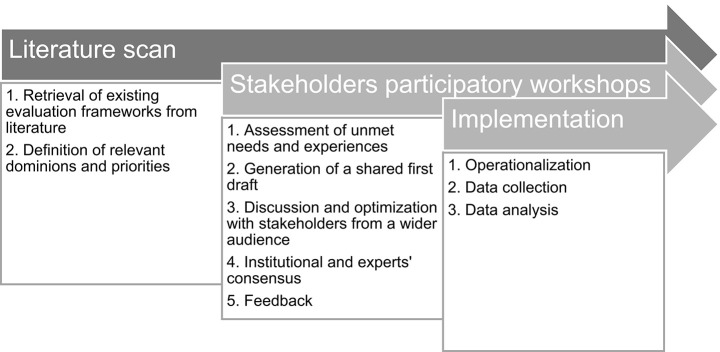
Summary of the methodological process for the framework development.

As mentioned above, the framework has been developed through a collaborative, iterative process consisting of the steps described below (Figure 1).

Particularly, a participatory approach was enabled in order to involve patients and healthcare professionals' representatives in giving their perspectives about the process of coproducing mental health services. This approach has the value to be grounded in the needs, issues, concerns, and strategies of the target populations in order to maximize the ability of the developed framework to be sensitive to real practice issues and priorities. This research approach has a collaborative nature that equitably involves healthcare professionals and patients as coresearchers in all the phases of the research process: all partners contribute their own expertise and ownership to reach shared decisions and to make the produced knowledge more rooted in the real clinical experience and able to be translated into the practice of service evaluation.

In particular, the participatory process was conducted with a two-step approach: (1) a narrative scan of relevant scientific evidences on co-production in mental health services and (2) a participatory process of evidence discussion, consensualization, and idea generation to reach a final consensus on a shared framework for evaluating co-production processes in mental health service development. Particularly, in this second step, four workshops were organized with the aim of discussing, refining, and gaining consensus on the developed framework and to systematically capture and incorporate their suggestions for the framework adaptation along the project. The workshops were organized with a bottom-up logic: starting from involving the direct project participants (project-related level), going up in order to gain a wide consensus from experts and decision makers (system-related level) outside the project.

#### Step 1. Preliminary Scanning of Relevant Literature

In parallel with the analysis of the experts' experiences and the needs of patients' experiences evaluation along the co-creation process, a narrative scan of scientific evidences related to the evaluation of co-creation practices has been conducted in order to identify standards and already existing guidelines in this field with a particular focus on mental health settings. This literature search was not systematic in its method, but rather narrative, and guided by both literature retrieved and discussion groups with project stakeholders. The decision to refrain from a systematic approach was due to the very nature of the research process itself, in which keywords were added or removed based on both a dynamic decision-making with project partners and an iterative process of familiarization with the literature, which lead to a gradual development of a conceptual framework using concepts embedded in the relevant lay, theoretical, and empirical literature; and a subsequent rearrangement of the data within the framework in order to insert each key dimension related to co-production evaluation in mental health settings. A snowball process of literature search was performed in order to “bottom-up” select relevant articles able to inspire and inform the subsequent process of analysis. A preliminary broad string of keywords was selected (i.e,. codesign or co-production or co-creation) combined with keywords indicating the clinical area of interest (i.e., mental health) and the type of articles sought (i.e., theoretical or methodological articles). The search was conducted across disciplines (medicine, nursing, social welfare, and the social sciences) accessing to different scientific databases (i.e., MEDLINE, Scopus, Web of Science, PsycINFO) and covered reports published in academic journals and patient- and carer-group publications, along with unpublished reports accessible through project-related professional and patient local networks. A selection of articles, particularly relevant at the conceptual level, was considered a milestone of this narrative scan of the literature and helped us in retrieving further similar and linked papers (i.e., from their references and citations). Articles were deeply read and conceptually analyzed to get insights related to the objectives of the study.

According to this process, the aim of this literature scan was two-fold:

- to identify already existing frameworks being used to assess the ability of patients in giving their preferences and in actively contributing in the definition of their therapeutic paths in mental health;
- to define objectives, priorities, and evaluation dominions for a comprehensive framework aimed at monitoring patients' ability to raise their preference and to collaboratively co-produce their therapeutic plan, which are the relevant constructs and dominions for co-production in mental health.

#### Step 2. Validation of Relevant Dominions by Stakeholder Participatory Workshop

Secondly, the research team engaged in a series of facilitated discussions with key stakeholders to develop and refine the framework, including defining domains, subdomains, and components, which also helped the research team to determine the point of saturation regarding domains and components, and to plan further searches of the literature. Particularly, domains considered influential for an effective service co-production process identified in the literature were discussed and reviewed by different stakeholder groups through subsequent collaborative workshops described as follows:

##### Workshop 1. unmet needs related to service evaluation practices in mental health settings

A first step toward the development of the framework for monitoring the co-produced, individualized care paths was intended to gain a better understanding of experiences, needs, issues, and current practices adopted by healthcare professionals involved in the Recovery.Net project sharing insights related to their own services. For this purpose, a focus group—defined as a “group interview” where “people are encouraged to talk to one another: asking questions, exchanging anecdotes, and commenting on each other's experiences and points of view” (27)—was carried out with 13 people (six males and seven females). In particular, six healthcare professionals, one expert patient, two expert consultants or academics, and four persons with administrative or supervisory duties (managers) were involved in the discussion, equally articulated for the three mental health settings involved in the project and differentiated per professional expertise. Table 1 summarized the participants who were involved in all the workshops. The focus group lasted 2 h and was articulated in the following two main sessions: (1) elicitation and collection of the professionals' experiences, practices, and instruments used for monitoring patients' experiences and for collecting their preferences in the definition of the therapeutic plan; (2) deep exploration of all the experienced issues and needs related to the assessment from both professionals and expert patients' views.

**Table 1:** Characteristics of participants involved in the participatory workshops.

**Workshop #**	**Expert patient/active user**	**Educator**	**Nurse**	**Physician**	**Administrative personnel**	**Psychologist**	**Consultant and academic**	**Other**	**Total**
#1 and #2	1	3	0	2	4	1	2	0	13
#3	14	26	15	9	3	9	3	15	94
#4	0	1	0	0	4	1	2	6	14
#5	2	6	3	3	3	1	4	1	23
Total	17	36	18	14	14	12	11	22	144

##### Workshop 2. generation of the first draft of the assessment framework

The conjoint synthesis of research evidence related to evaluation priorities and assessment domains to be included in the monitoring framework was presented and deeply discussed in a second workshop involving the same stakeholders. In particular, the workshop was articulated in the following two research phases. (1) Initially, all experts had the opportunity to raise their concerns and interpretation regarding evidences collected and to contribute in building a first evaluation framework draft. This session was an important chance to fertilize clinical experience with insights from the international literature and to jointly agree on evaluation priorities. (2) The workshop ended with the stakeholders' agreement on the framework structure, domains, and first draft. A first draft of the framework, thus, was developed to be presented for later discussion and refinement.

##### Workshop 3. discussion and optimization of the framework

A third workshop was organized, including a larger group of stakeholders, in order to involve a wider range of experiences and insights also from patients and healthcare professionals who did not participate at the definition of the first draft. Furthermore, not only stakeholders belonging to the Recovery.Net project were involved in this workshop but also clinicians and patients' representatives coming from other settings, although similarly involved in co-creative efforts related to the definition of individual therapeutic plans. In total, 94 persons (32 males and 62 females) participated to the workshop. Among these, 14 were expert patients or active service users (see Table 1 for details). This more inclusive workshop was structured into three different moments: (1) a first part was structured as a lecture about the concepts of recovery, patient engagement, and co-production with particular reference to mental health care; after the introduction, the framework was presented; a Q&A session followed. (2) In the second part of the day, smaller discussion groups were formed. Every single group had a moderator and the instruction to discuss a specific aspect of the framework, of its constituents, and, in general, its applicability in healthcare services. (3) Finally, results from the discussion groups were discussed in plenary in order to share alignment and misalignment among the groups' proposals. At the end, consensus was reached about shared priorities and dominion for the final version of the framework.

These three workshops allowed to critically revise and refine the first draft of the framework, thus promoting a further definition of the core elements featuring the framework structure.

##### Workshop 4. consensualization and transferability of the framework

A final workshop was organized in a Regional institutional setting, involving key stakeholders, opinion leaders, and policy makers experts in co-productive practices in mental health. The aim of this workshop was to present the final version of the framework to an expert audience to gain a final consensus and to discuss its transferability to clinical settings different from the piloting setting provided by the Recovery.Net project. Among the 14 participants to this workshop (four males and 10 females), international experts as well as experts from other regions in Italy were involved in this final workshop as well in order to challenge the achieved framework and to further enrich it with insights from different practices and experiences. General availability and involvement of these key stakeholders in the application of the framework was assessed as well and discussed in order to develop shared guidelines for its application in real mental health settings.

##### Workshop 5. definition of implementation guidelines

A final workshop was then organized with the management group of the project and with stakeholders involved in the Recovery.Net project to report the results of this process and the consensus gained on the framework and to develop shared guidelines for the framework implementation in daily clinical practice. Twenty-three persons (four males and 19 females) participated in this final workshop (see Table 1 for details).

### Workshop Data Analysis

Data analysis involved three phases: identifying the themes, developing a framework, and confirming the framework. We coded and organized the data and abstracted, illustrated, described, and explored the emergent themes using thematic analysis. At the same time, content analysis was conducted to retrieve concepts and insights from relevant literature about health services co-production to integrate and extend the emergent conceptual framework. The framework was finally reviewed by the research partners belonging to the study project and preliminarily implemented.

## Results

**Figure 2:**
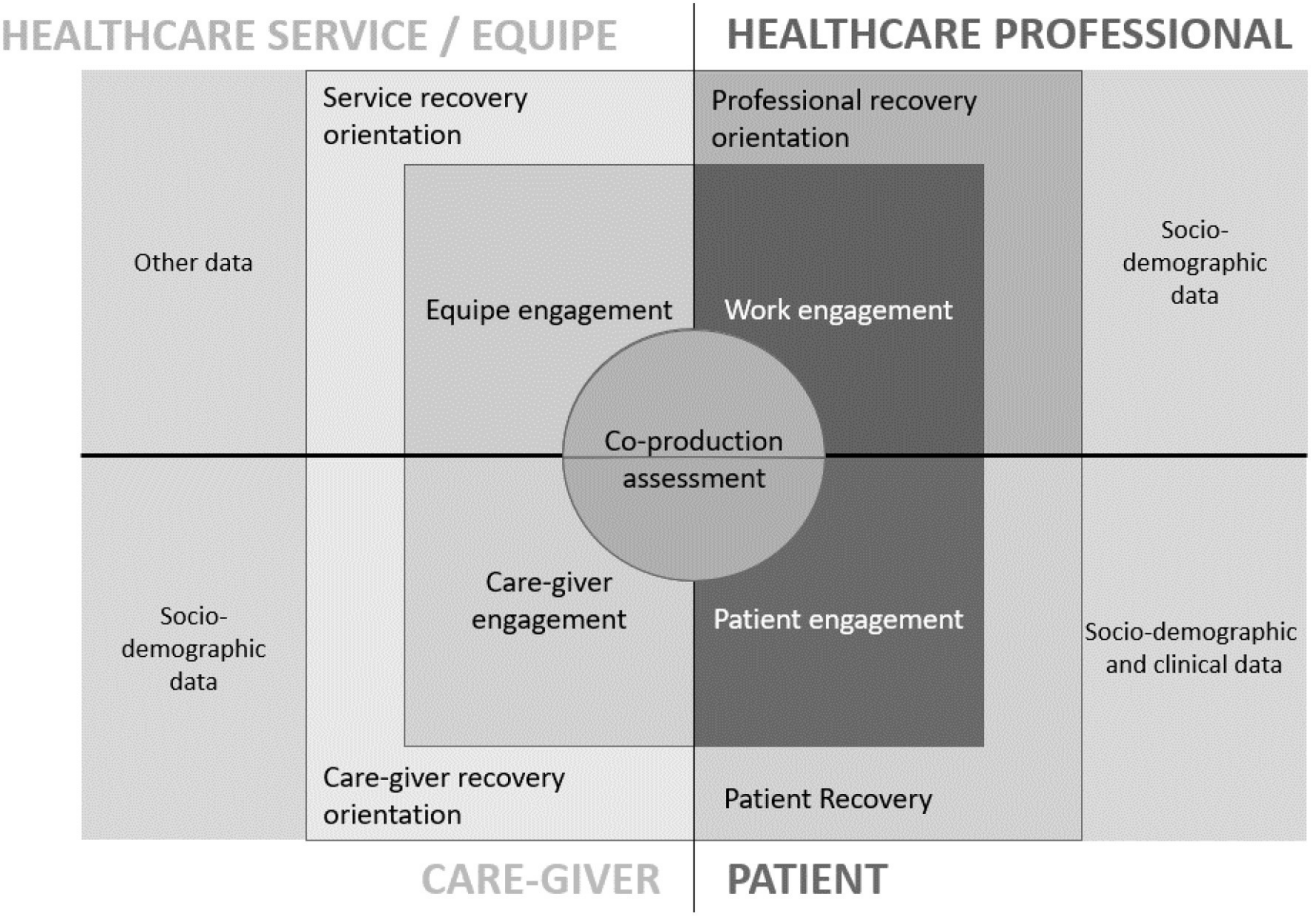
The co-creation compass: detailed description of the framework elements.

According to the results of both the literature scan and the participatory workshops, the evaluation framework (COCO framework) for monitoring patient preference collection when co-producing medical pathways was drafted (Figure 2)

Following the main evidence and issues raised by the research process that informed the nature and the characteristic of the framework.

### The Need for Shared and Practice-Oriented Evaluation Standards

We revised existing literature on co-production assessment, with particular attention to mental health settings, with a narrative—rather than systematic—approach. Despite the fact that the approach of co-production has—in the last decades—spread out in different fields (28) and despite the fact that scientific literature is consistent in claiming that, in mental health, co-production of healthcare services, significant relationships between patients and healthcare professionals, and recognition of one's own contribution toward his/her healthcare plan play an important role in determining health outcomes, quality of life, patient satisfaction, service innovation, and cost savings (19, 29), our literature search was unable to retrieve an evaluation framework for co-production which has a wide consensus in scientific community. While it was possible to retrieve some instruments intended to assess co-production in healthcare services (30), they seem to lack a solid, peer-reviewed, reference framework that vouches for its replicability and usefulness in various contexts and, in particular, in mental health. Nevertheless, from the critical revision of these instruments and of the relevant literature (31–34), five dominions critical for a successful co-production of healthcare services were identified, namely, (1) participation as a value, (2) shared decision-making, (3) healthcare service co-construction, (4) healthcare service as a catalyst for change, (5) users as assets.

Additionally, according to the “house of care” model, proposed by Coulter et al. (35), patients' and healthcare professionals' engagement (in their own health and work, respectively) seem to be another fundamental ingredient for the successful development of an individualized, co-produced, care plan.

Furthermore, although the well-known relevance of measuring what the patients' preferences are, and the implementation of this practice in some clinical settings of PROMs and of PREMS to assess the patient priorities when using services (35–38), still these experiences of measurement are at their infancy and jeopardized in their level of implementation, particularly in the Italian context. Many problems still persist in terms of no shared guidelines for evaluation frameworks dedicated to the mental health settings, no shared procedures of application and data analysis, and particularly, a very simplistic approach to such data analysis, not considering the multilevel determinants which impact on patients' experiences of service co-production from the perspectives not only of patients but also of their health care professionals.

Similarly, also in current clinical practice of the stakeholders interviewed, no shared guidelines and instruments were retrieved for assessing patients' input elicitation. The first workshop with the stakeholders revealed that most services were implementing customer satisfaction surveys for the patients and their relatives and that only the clinical stature of the patients was monitored systematically. Some services had also implemented sporadic analysis of healthcare professionals' work-related well-being and work satisfaction. However, the most common issue expressed was the lack of a reference framework; moreover, it was stated that “*there is not a ‘culture’ for evaluation”* and that often data and feedbacks collected through their surveys “*didn't have much impact on their actual practices*. ” Finally, they reported a lack of sustainability: since the perceived impact of those monitoring practices was low, it was difficult to systematically carry them out, causing many missing data, in particular during the posttreatment assessment. Professionals and expert patients discussed the possibility that an assessment framework for a co-produced path should assess not only patient's satisfaction with the service but even other aspects of his/her experience. The group also showed interest in an assessment comprising not only the patient's experience but also the experiences of those surrounding him/her (e.g., caregiver). The developed framework (COCO), according to these suggestions, adopted a multi-perspective evaluation of the co-productive process in order to get insights about the alignment or misalignment of the stakeholders' experiences. Furthermore, all the materials being used by the healthcare services were shared among the members of the group for revision and to creatively base on these experiences the development of the COCO framework.

Furthermore, experts and patients involved in the workshop raised their voice to advocate for the need of developing a new framework able to systematize current practices in the assessment of patients' contribution to co-creation of therapeutic plans. Particularly sought was the definition not only of the indicators and scales to be included in the evaluation, but also of the dedicated assessment moments to be planned, the stage of the co-creative process at which to implement them, and with what comparison of the achieved results. Furthermore, cues about ways to influence the service provided and the course of the therapeutic paths with the assessment framework were sought as well.

A shared need expressed by experts and patients interviewed was a better systematization of the existing attempts to assess a co-productive process. However, they also expressed concerns about the risk of increasing bureaucracy burden on the clinical routines and raised the point of potential cultural and organizational resistances against changes in daily routines. For these reasons, the COCO framework adopted both new indicators and just used ones in order to facilitate its implementation into practice.

### The Quest for a Multi-Dominion Approach

Even though during the initial focus group emerged that the assessment of the patient was mainly focused on clinical variables and on his/her satisfaction, the scan of the literature revealed that there are other relevant variables that need to be addressed and considered during the evaluation of a patient that is following a co-productive care path. Moreover, the analysis of the literature about co-production evaluation and the insights collected through the participatory workshops revealed that there are several aspects to be considered when implementing process healthcare service co-production. Three of them seem to be present in the scholarly relevant literature and have been finally involved in the COCO framework:

◦ *Orientation to service co-production* Implementing the co-production paradigm in healthcare requires a positive attitude toward a shared design of services by patients, their families, and healthcare professionals (as individuals and as individuals in a team). Following the principles of co-production may require an organizational change in the balance of power among the healthcare actors and a broader culture shift in service development and provision. It involves viewing interactions as reciprocal by shifting the focus away from solely delivering services and toward facilitating and enabling people to access services and resources. It also will involve acknowledging risks and creating a plan to manage them. In particular, it is crucial to evaluate at these different levels how co-production is perceived in terms of the actual enactment of shared decision-making practices, participation as a value for the stakeholders, mutual acknowledgment of the skills and knowledge of the different stakeholders, and stakeholders' actual perception of being engaged in co-production.
◦ *Engagement as an enabling factor* Shared decision-making and co-production are concepts that are increasingly used in the context of managing long-term conditions, such as mental diseases. Both concepts recognize that improvements in health and well-being outcomes cannot be driven by health professionals alone but require the *active engagement of patients*. This is because the effective management of long-term conditions is largely dependent on what people do day by day for themselves, rather than on professional clinical interventions. Secondly, only a patient can really be aware about his or her own priorities and preferences, and for care to be effective, it must be shaped around these priorities. Moreover, family caregivers are a fundamental support for patients along their recovery journey, and their presence can also sensitize patients in raising their voice in the co-creative process. The *level of engagement and participation of family caregivers*, thus, is a further important factor to be monitored in the co-production process in order to assess the conditions which may sustain or hinder patients' ability to contribute and give input along the process. Besides patient and family engagement, scientific literature and clinical practice suggest the cruciality of the *healthcare professionals' commitment to participatory practices*. Studies (39) raise concerns related to the impact of healthcare professionals' burnout and fatigue on their effective ability to motivate patients in being engaged and give input in co-creation practices. The level of work-related well-being, engagement, or (on the contrary) burnout and healthcare professionals' orientation toward collaborative healthcare practices (39, 40) might impact on their approach to the patients and their openness to co-creation. A top-down health policies' imperative to embrace co-creation and to sought input from patients in clinical research, thus, could be perceived by healthcare professionals as a further professional duty and as a limitation to their clinical decision-making about treatment, instead of a way to make their practices more effective and sustainable. Thus, the level of healthcare professionals work engagement and commitment to co-creation is an important factor impacting their actual ability to collect patients' input (41, 42).
◦ *Recovery orientation* Being the treatment of mental health concerned, the goal of recovery is consensualized as a primary endpoint. Mental recovery is defined as the ability of the patient to become resilient with the clinical condition and to acquire motivation, skills, and competences to change his/her behaviors and attitudes toward his/her life. Thus, a new monitoring framework aimed at evaluating the co-production process in which patients provide input about their care pathways should take into consideration the recovery process of the patients, together with the experience and attitude toward patients' recovery of the other significant stakeholders (i.e., healthcare professionals and informal caregiver) along the care process. Also in this case, it is necessary to evaluate the orientation toward recovery in the perspective of all the actors participating in the codesign of the care practice (i.e., patients, families, healthcare professionals, and the care team as a whole). When orientations are not fine-tuned, this might result in invisible barriers for the implementation of innovation in service practices (43).

### The Need for a Multi-Stakeholder Evaluation

In the workshops, experts and patients raised their voice against an oversimplification of the monitoring concept. This led to the proposal of a multi-stakeholders' approach, aimed to not only assess the patient's experience but also put his/her experience in relation to that of other stakeholders involved with his/her care. The basic assumption was, indeed, that co-production and patients' input elicitation is actually possible only if both healthcare professionals and patients are keen to collaborate and to introduce this organizational change in their current practices.

On this basis, from the results of the multistep process described above, four principal stakeholders were identified:

The patient him/herself;

- His/her family caregiver, defined as the relative, friend, or person that informally takes upon him/herself the responsibility of the patient's care;
- The healthcare professional of reference: every personalized care path is carried out with the involvement of more than a single individual, and different professionals contribute to the success of the care path. Anyway, it was possible to identify a professional (either a physician, a psychologist, a nurse, or a professional educator) for each patient who is responsible for his/her care inside the Service more than the others and who is seen as his/her “referent”;
- The healthcare service: different healthcare services—defined as different hospital structures, social cooperatives, etc.—involved in Recovery.Net. Since such different services are often different in their practices and in the environment that they provide and in which the patient is taken care of, it was perceived as a fundamental to assess the environment and the climate in which the patient him/herself is included.

Further than the need to seek evaluation from multiple actors involved in the individualized therapeutic pathway, a claim was made to enhance the comparison of such evaluations. Particularly, to generate mirroring occasions among the contemporary experiences of patients, their clinicians, family caregivers, as well as the complete healthcare team was indicated as crucial.

We have argued above how it is important to avoid a simplistic notion of clinical value for therapeutic intervention and, on the contrary, to opt for a notion of value as situated and co-created on the basis of the psychosocial experiences and expectations of the key actors involved in the decision-making about treatment. Doctor and patient have their own range of individual values: hence, it might be quite unrealistic to imagine that they always assign the same meaning to aspects such as health and care.

This is particularly true in mental health, where the value of pharmacological performance often sought by clinicians may be experienced in spite of severe side effects or of impact on patients' daily life, thus being perceived with less enthusiasm by patients (44). Cocreating value requires the collaborative activities of actors involved in the service exchange (45), which are dependent on the capabilities and resources available to the provider (e.g., expertise, technology) and the consumer (e.g., knowledge, experience) as the two relevant parties. When talking about engagement in value-based healthcare, it is crucial to consider at least two main actors involved in the care pathway: the patient and the doctor. Physicians are on the front lines of this change: their role in realizing a value-based healthcare is pivotal in achieving this end. However, scholars also advocate for the importance of engaging patients in the shared decision-making process about treatment options in order to give voice to their health priorities and expectations. This implies the importance of achieving a good concordance between patients and clinicians about what is value in healthcare and how this should be determined, although this is not often achieved due to the lack of communication and of mutual understanding of the two actors. So, all these reflections put in discussion the real actualization of a value-based healthcare because of these barriers at different levels. On the contrary, we hypothesized that greater alignment about the “expected value” of healthcare processes between patients and their healthcare providers might actually enable a co-produced high-value health care service in the aim of “good health for all.”

### Co-production Compass: Consensualized Framework and Implementation Guidelines

After having designed and discussed with the key stakeholders the theoretical assumptions of the framework, the two final workshops were dedicated to (1) consensualize a final architecture of the COCO and (2) draft related guidelines for its implementation.

In particular, four further steps have been considered and elaborated: (1) *framework architecture*; (2) *dominions' operationalization* (which measures/indicators to assess the different dominions planned by the framework?); (3) *strategy of assessment* (when applying the assessment framework along the process of service co-production?); (4) *analysis outcomes* (which analysis outcomes should be expected from the application of the COCO framework?).

#### Frameworks Architecture

In order to concretely answer to the shared expectations that emerged in the workshops, it was proposed that the framework allowed to monitor simultaneously experiences and evaluation of the four groups of stakeholders identified (i.e., patients, healthcare professionals, caregivers, and healthcare team) on the same three key dominions (i.e., co-production, engagement, recovery) in order to allow a complete and articulated vision on the co-production process enhanced in the service.

The simultaneous and systematic monitoring of such evaluations from the different stakeholders would enable continuous triangulation of data and mirroring between key stakeholders on the same crucial experiences (for instance, patients and their healthcare professionals, patients and their caregivers, healthcare professionals and their team, and so on), as graphically proposed in the diagram of Figure 2.

In other words, the monitoring framework could be implemented in the healthcare organization through the administration of the multi-perspective assessment tools to the different stakeholders to gain “mirrorable” data.

**Figure 3:**
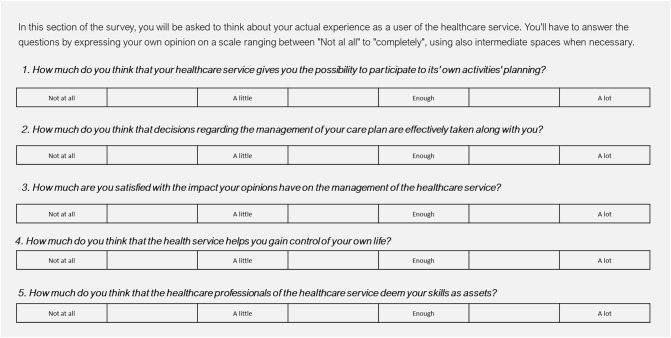
Items for measuring patient's input in co-creation processes.

Furthermore, the three main dominions discussed above were included in the framework architecture and operationalized through the identification of key indicators suggested from both the scientific literature and the experts' opinion (see Figure 3 for more details).

In particular, since no specific tool regarding the co-production domain in mental health was found which could suit our needs, we developed a new scale, composed of 10 items (see Figure 3 for the list of items) addressing the key dimensions of service co-production (the actual enactment of shared decision-making practices; participation as a value for the stakeholders; mutual acknowledgment of the skills and knowledge of the different stakeholders; stakeholders' actual perception of being engaged in co-production). Items were developed *ad hoc* together with stakeholders involved in the workshops and on the basis of tentative experiences developed in some of their settings. This scale is yet to be validated.

Engagement—the second key domain identified in the previous phases—was operationalized throughout the adoption of specific engagement-related scales retrieved from the scientific literature and demonstrated to be reliable for assessing the level of engagement of the different stakeholders involved in the healthcare process [i.e., Patient Health Engagement Scale (PHE-S®) (46); Caregiving Health Engagement Scale (CHE-s) (47); Utrecht work engagement scale (48)].

**Figure 4:**
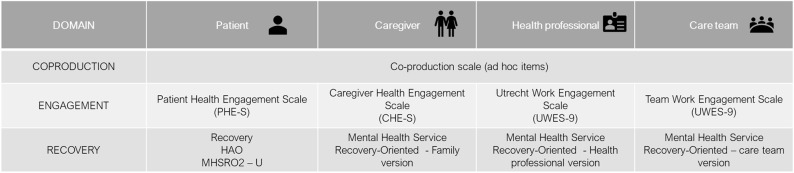
Domains' operationalization.

Finally, to operationalize the domain of Recovery, the scales just applied in the usual care was adopted by the framework in order to be more easily applied in the clinical practice (see Figure 4 for an overview of domains' operationalization).

#### First Implementation Guidelines

From a strategical point of view, it is fundamental to establish the timing of the measurement framework application. Beyond the standard turning points of the patients' care, it could be necessary to apply the assessment framework in not standardized moments that might vary depending on the patients' disease and illness history, the setting of care, the sociocultural context the patient belongs to, and the patients' ability to self-report. Moreover, the healthcare organization that wants to adopt this framework to assess pathways of service co-production should consider that assessment could follow two main logics. On one side, a “linear logic” might suggest to administer measures in crucial turning points of the care process (i.e., diagnosis, treatment decision-making, hospital discharge, clinical follow-ups.); on the other hand, a “processual logic” might encourage organizations to administer measures when critical events occur in the disease course. These events could be both clinical-related (i.e., new symptoms, disease relapse, unexpected new diagnosis.) and patients' context-related (i.e., caregivers' disease or death, changes in the patients' psychological status). All these variables, indeed, could have an impact on the patient's availability to engage in a co-production process.

Regarding the expected monitoring outcomes that the framework could make possible for organizations adopting it, two main results could be expected:

- both punctual and longitudinal data emerging from the repeated administration of the assessment framework along the co-generation of the care plan could be obtained;
- moreover, the multi-stakeholder nature of the COCO framework allows obtain “mirrorable” data from the different stakeholders, as mentioned at point (1) of this paragraph (i.e., patients, caregivers, health professionals, care team) involved in a care process informed by a co-productive paradigm.

The framework allows not only to assess the patients'/caregivers' and the professionals' experiences separately but also to integrate them and to see how much they are aligned. This is carried out on two different levels:

- First, by keeping track of the links existing between the different stakeholders: between patients/caregivers and professionals and between professionals and the care team;
- Second, by asking professionals and caregivers not only to report their own experience but also to report what they thought their patients' experience was like: this allows for the generation of “mirrorable” data, in which the same object—namely, the patients' experience—is assessed from four different points of view. The key data of this approach, actually, is not the experience *per se* but the degree of alignment/misalignment of the different perspectives.

**Figure 5:**
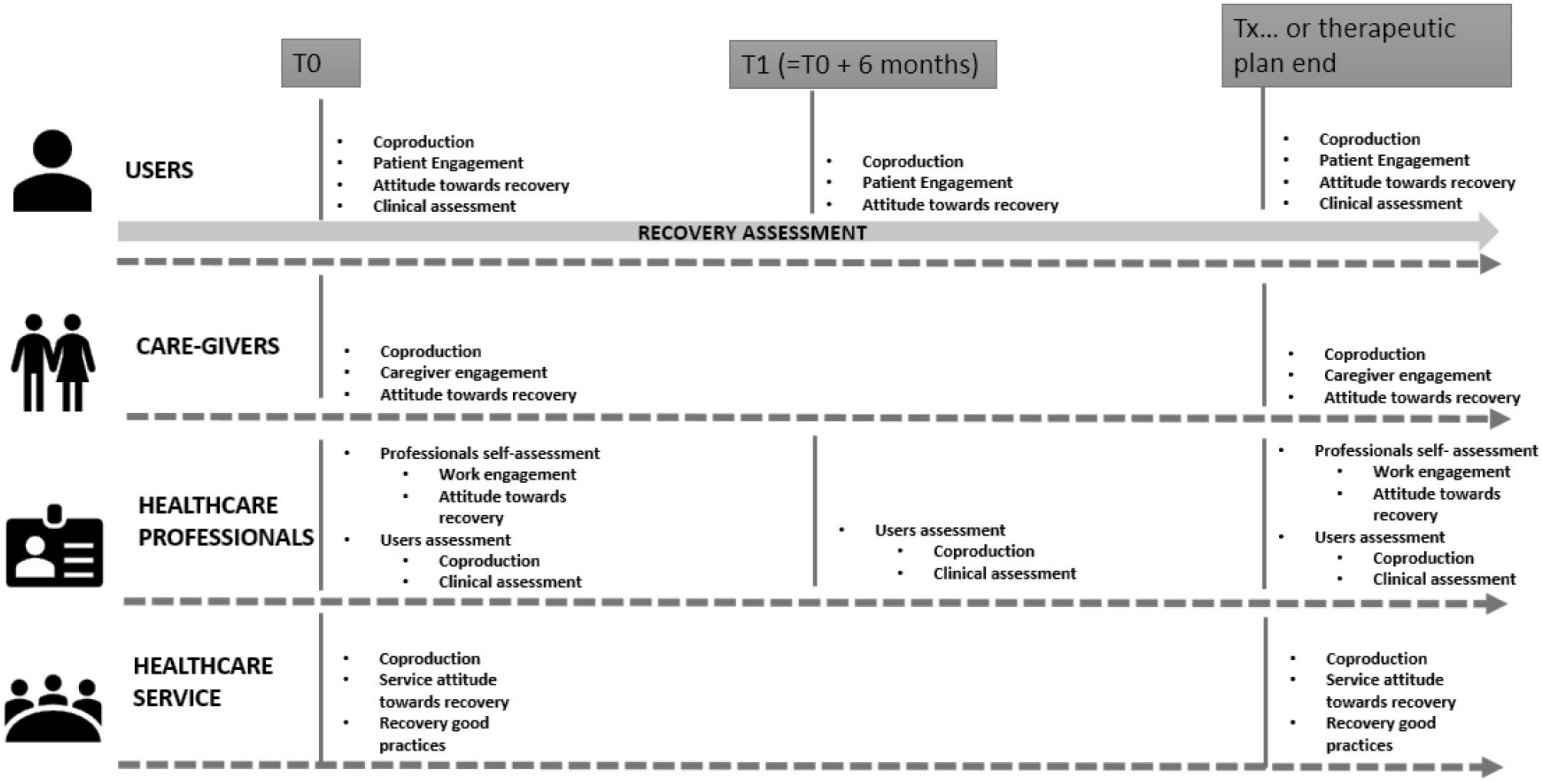
Example of timings for the framework implementation.

The Recovery.Net project developed a specific guideline related to the timing of the framework implementation as described in Figure 5.

## Discussion

Although co-production requires very huge efforts by both healthcare systems and patients to be fully implemented because of the related efforts in the generation of adequate settings and processes, literature shows encouraging results in improving patients' engagement and quality of their experience (29). However, the lack of a systematic approach may hamper the implementation of such organizational changes and make the accomplishment of such results particularly hard to obtain.

Tools and protocols devoted to elicit patients' preferences already exist and are reaching a good level of consensus within the scientific and clinical community; nevertheless, the issues of how to measure and monitor the effectiveness of such tools in guaranteeing the inclusion of patients' preferences in the therapeutic planning appear to be fairly ignored (49). The literature on co-production in mental health generally focuses on the outcomes/contents of the co-produced activities, paying less attention to people's motives to co-produce and to the process of co-production in practice. Moreover, evidence from the field suggests the opportunity to pursue a “multi-stakeholder approach” when developing co-production projects aimed to deliver a mental health service which is actually able to face inequalities in mental health service access and provision.

Based on these premises, in this paper, we described the COCO, a new proposed framework to monitor the extent to which patients, together with other stakeholders, are involved in co-productive processes in mental health.

This conceptual framework, even though still needing evidence from first piloting exercises, appears innovating in its extent to systematize the monitoring of crucial factors enabling co-production in mental health care. First, this framework aims at offering a better systematic approach to co-production assessment and monitoring in healthcare in order to allow a more effective implementation and use of elicited data.

Moreover, it appears particularly innovative in its multi-stakeholders and multi-dominion design. The idea of collecting experiences and evaluations from different stakeholders contemporaneously on the same co-production process may enable a better rigorous triangulation of perspectives; furthermore, it can enhance clinical practices by highlighting areas of alignment, misalignment, and mismatch in the way different stakeholders attribute values and meanings to shared experiences of co-production instead of just focusing on the single perspective of the user.

According to the complex and fluid nature of co-production process, the framework aims at balancing a qualitative clinical vision of co-production, by taking into account subjective evaluation and psychological enhancers of such experiences such as the concept of engagement (1, 2) with a method and a protocol to systematically transform such qualitative and subjective nuances in a synthetic score.

Moreover, the framework has the value of embracing the complexity of patients' input elicitation in healthcare by considering it as the result of a dynamic process where motivational factors of the factor, together with other contextual and relational factors (i.e., relation with the healthcare provider or with the patients' family caregivers) may enhance or hinder such process. Evaluating the effectiveness of co-production, thus, needs a longitudinal perspective and a multilevel scope of analysis in order to grasp the evolutionary pathways of co-production phases and dynamics.

Finally, an additional value of the COCO relies on its developmental process itself based on the continuous comparison within patients, healthcare professionals, and real settings and aimed at developing tools and models of evaluation really adoptable in the correct clinical practice of the settings involved in the process. This participatory process of the framework development is a potential guarantee of acceptability of this new framework in the healthcare settings and by their patients.

Due to such characteristics of the framework and of the process which lead to its development, the COCO presents potentialities of application also to other healthcare sectors to magnify the psychological processes implied in the co-creation dynamics.

## Limitations

Although promising, this framework needs further exploration; in particular, some of the tools used or developed for the implementation of the assessment framework need further validation and fine-tuning. Data collected during the duration of the Recovery.Net project will furnish the first evidence toward these regards. Furthermore, future lines of research may be devoted to assessing the exportability and sustainability of this framework to other healthcare settings and clinical areas, with larger and more representative samples of mental health patients or other chronic patients. Finally, a cultural and organizational adaptation of the framework may be envisaged to explore if additional enabler or hindering factors to co-production process need to be concerned and included in other healthcare systems.

## Data Availability Statement

The datasets generated for this study are available on request to the corresponding author.

## Ethics Statement

The studies involving human participants were reviewed and approved by Ethics Committee Spetali Civili di Brescia. The patients/participants provided their written informed consent to participate in this study.

## Author Contributions

GG and SB have designed the study protocol, supervised the research process, and given creative and theoretical input to its results. GG has led the project team from ideation to implementation of the study. SB, LP, and GG have moderated the workshops and first drafted the paper. LP has conducted the literature scan. FL revised the paper and gave input to refine its conclusions. All authors contributed to the article and approved the submitted version.

## Conflict of Interest

The authors declare that the research was conducted in the absence of any commercial or financial relationships that could be construed as a potential conflict of interest.
